# Frequency-coded patterns of sympathetic vasomotor activity are differentially evoked by the paraventricular nucleus of the hypothalamus in the Goldblatt hypertension model

**DOI:** 10.3389/fncel.2023.1176634

**Published:** 2023-08-22

**Authors:** Jean Faber, Maycon I. O. Milanez, Cristiano S. Simões, Ruy R. Campos

**Affiliations:** ^1^Neuroscience Division, Department of Neurology and Neurosurgery, Escola Paulista de Medicina, Universidade Federal de São Paulo, São Paulo, Brazil; ^2^Cardiovascular Division, Department of Physiology, Escola Paulista de Medicina, Universidade Federal de São Paulo, São Paulo, Brazil

**Keywords:** neural frequency code, hypertension, paraventricular nucleus of the hypothalamus, renal sympathetic activity, splanchnic sympathetic activity, losartan, bicuculline

## Abstract

**Introduction:**

The paraventricular nucleus of the hypothalamus (PVN) contains premotor neurons involved in the control of sympathetic vasomotor activity. It is known that the stimulation of specific areas of the PVN can lead to distinct response patterns at different target territories. The underlying mechanisms, however, are still unclear. Recent evidence from sympathetic nerve recording suggests that relevant information is coded in the power distribution of the signal along the frequency range. In the present study, we addressed the hypothesis that the PVN is capable of organizing specific spectral patterns of sympathetic vasomotor activation to distinct territories in both normal and hypertensive animals.

**Methods:**

To test it, we investigated the territorially differential changes in the frequency parameters of the renal and splanchnic sympathetic nerve activity (rSNA and sSNA, respectively), before and after disinhibition of the PVN by bicuculline microinjection. Subjects were control and Goldblatt rats, a sympathetic overactivity-characterized model of neurogenic hypertension (2K1C). Additionally, considering the importance of angiotensin II type 1 receptors (AT1) in the sympathetic responses triggered by bicuculline in the PVN, we also investigated the impact of angiotensin AT1 receptors blockade in the spectral features of the rSNA and sSNA activity.

**Results:**

The results revealed that each nerve activity (renal and splanchnic) presents its own electrophysiological pattern of frequency-coded rhythm in each group (control, 2K1C, and 2K1C treated with AT1 antagonist losartan) in basal condition and after bicuculline microinjection, but with no significant differences regarding total power comparison among groups. Additionally, the losartan 2K1C treated group showed no decrease in the hypertensive response triggered by bicuculline when compared to the non-treated 2K1C group. However, their spectral patterns of sympathetic nerve activity were different from the other two groups (control and 2K1C), suggesting that the blockade of AT1 receptors does not totally recover the basal levels of neither the autonomic responses nor the electrophysiological patterns in Goldblatt rats, but act on their spectral frequency distribution.

**Discussion:**

The results suggest that the differential responses evoked by the PVN were preferentially coded in frequency, but not in the global power of the vasomotor sympathetic responses, indicating that the PVN is able to independently control the frequency and the power of sympathetic discharges to different territories.

## 1. Introduction

The sympathetic nervous system (SNS) plays a fundamental role in maintaining body homeostasis. The sympathetic overactivation is frequently associated with pathological conditions and their severity (Guyenet, 2006; Carnagarin et al., 2018). Vasomotor sympathetic overactivation, for instance, affects the cardiovascular system, leading to hypertension, chronic kidney disease, and heart failure (Carillo et al., 2012; Campos et al., 2015; Nishihara et al., 2017).

The reciprocal communication between the peripheral organs and structures of the Central Nervous System (CNS), including areas from the brainstem, basal forebrain, and spinal cord involved in the control of vasomotor sympathetic activity, plays a pivotal role in the regulation of cardiovascular function (Dampney, 1994, 2016). The activation of specific brain areas, however, can lead to differential sympathetic vasomotor responses and even opposing effects in different target organs under both physiological and pathological conditions (McAllen and May, 1994; Rumantir et al., 1999; Salo et al., 2006; Ramchandra et al., 2012; Esler et al., 2018). The stimulation of the parvocellular neurons of the paraventricular nucleus of the hypothalamus (PVN), for example, may simultaneously trigger the reduction of the renal sympathetic nerve activity (rSNA) and the increase of the activity of the splanchnic, cardiac, and adrenal sympathetic nerves (Deering and Coote, 2000). Given such distinct outcomes triggered by the same stimulation, it seems that the PVN activity is differentially coded downstream, depending on the nerve outflow and the target organs.

Previous studies investigating the differential activity patterns in the sympathetic nerves so far have mostly focused on the global energy of the signal, lacking a better understanding of its distribution over the frequency range (Wehrwein and Barman, 2014; Barman, 2020). The technical evolution of both electrophysiological recordings and signal analysis in the last few decades, however, has shown that global energy analysis alone is not enough to link sympathetic nerve activity to the genesis and development of hypertension (Wehrwein and Barman, 2014). Evidence has emerged suggesting that relevant information is coded in the spectral features of the sympathetic discharge (Barman et al., 1992, 1995; Chang et al., 1999; Milanez et al., 2020b). The firing frequency of the discharges recorded in the vasomotor sympathetic nerves reflects both their central generation and entrainment by the pulsatile input from the arterial baroreceptors while their amplitude is related to the number of recruited fibers (Guild et al., 2010). There is considerable evidence that stimuli independently controlled and affected the two parameters, such as inputs from chemoreceptors and baroreceptors (Guild et al., 2010), although their central organization by the CNS is still poorly understood.

The PVN contains premotor neurons involved in the control of sympathetic vasomotor activity and it has been described that the imbalance of inhibitory versus excitatory inputs in the PVN triggers sympathetic overactivation and hypertension (Martin et al., 1991; Bergamaschi et al., 1995; Allen, 2002; Barman, 2020; Milanez et al., 2020b). The disinhibition of the PVN through local microinjection of the γ-aminobutyric acid (GABA)-ergic antagonist bicuculline triggers both a robust hypertensive response and changes in the sympathetic vasomotor activity pattern to the renal, splanchnic and lumbar territories, generating low-frequency bursts unsynchronized to the cardiac cycle (Kenney et al., 2001). On the other hand, local GABAergic agonism significantly reduces rSNA (Zhang and Patel, 1998). The changes triggered by bicuculline are, in part, dependent on the activation of Angiotensin II type 1 (AT1) receptors (Milanez et al., 2018, 2020a,^2021^), as the microinjection of the AT1 receptor antagonist losartan into the PVN has been reported to blunt the sympathoexcitation evoked by local GABAergic antagonism (Chen and Toney, 2003). GABAergic neurons are, thus, crucial for the PVN cardiovascular function through peripheral sympathetic discharges. In the present work, we took advantage of this approach in a Goldblatt two-kidney, one-clip (2K1C) rat hypertension model to advance our understanding on how the PVN disinhibition influences the sympathetic vasomotor activity in normal and hypertensive states. For that matter, we addressed the territorially differential changes in the frequency parameters of the basal and bursting activities of the renal and splanchnic (sSNA) sympathetic nerves. Additionally, considering the local crosstalk between angiotensinergic and GABAergic systems, we investigated the effects of the systemic administration of losartan on the induced responses by bicuculline into the PVN and increased sympathetic nerve activity in 2K1C rats.

## 2. Materials and methods

### 2.1. Animals and ethical approval

All procedures were approved by the Ethics in Research Committee of the Escola Paulista de Medicina – Universidade Federal de São Paulo (protocol no. 8724270715/15) and performed in accordance with the guidelines of the National Institute of Health. Male Wistar rats (250–350 g) were housed in group cages under controlled temperature of 23°C and 12/12 h light/dark cycle with free access to food and water.

### 2.2. Induction of the Goldblatt renovascular hypertension and losartan treatment

Ten 5-week-old animals were anesthetized intraperitoneally with ketamine (80 mg/Kg) + xylazine (10 mg/Kg). A silver clip (gap width = 0.2 mm) was carefully implanted around the left renal artery for the induction of the two-kidney, one-clip (2K1C) Goldblatt renovascular hypertension. All animals had hypertension successfully induced. Five weeks later, half of them were treated with losartan (30 mg/kg/day) delivered by oral gavage for 7 consecutive days, as previously described (Nishi et al., 2013).

### 2.3. Experimental and control groups

Experiments were performed 6 weeks after the implantation of the clip, which corresponds to a temporal peak in the sympathetic neurogenic mechanisms underlying the Goldblatt hypertension in the PVN (Campos et al., 2015). The animals were divided in three groups: Goldblatt hypertension-induced rats (2K1C, *n* = 5); Goldblatt hypertension-induced rats treated with losartan (2K1C + LOS, *n* = 5); and age-matched control rats not submitted to Goldblatt surgical procedure (CTRL, *n* = 5).

### 2.4. Recording of mean arterial pressure and heart rate

All animals were independently submitted to ketamine (80 mg/Kg) + xylazine (10 mg/Kg) anesthesia and had the femoral vein catheterized for direct recording of mean arterial pressure (MAP) and heart rate (HR), that were carried out in conscious rats after surgical recovery (approximately 24 h) (PowerLab – AD Instruments, Australia). Average values were obtained by continuously recording for 10 min before urethane intravenous administration.

### 2.5. Electrophysiological recording of renal and splanchnic sympathetic nerve activity and microinjection procedure

Rats were anesthetized with urethane (1.4 g/kg, i.v.). The left renal and splanchnic nerves were retroperitoneally exposed and placed on bipolar silver electrodes, and once the conditions for electrophysiological recording were established, both the nerve and the electrode were covered with paraffin oil.

The animals were then placed in a stereotaxic apparatus (David Kopf, USA) for the microinjection procedure and electrophysiological recording. The PVN was located 1.8 mm caudal to the bregma, 0.5 mm lateral to the midline, and 7.8 mm deep from the dorsal medullary surface (bite bar = 3.6 mm) (Paxinos and Watson, 2006).

The baseline signal (BAS condition) from the activity of both nerves was amplified (gain 20 K, NeuroLog, Digitimer, Welwyn Garden City, Hertfordshire, UK), bandpass filtered (100–1,000 Hz), and recorded for 10 min using a PowerLab data acquisition system (AD Instruments, Sydney, NSW, Australia) for subsequent analysis.

After basal activity recording, the GABAa receptor antagonist bicuculline (400 pMol in 100 nl) was bilaterally microinjected into the parvocellular region of the PVN using glass micropipettes with tip diameters of 10–20 μm connected to a nitrogen pressure injector (MicroData Instruments Inc., USA), as previously described (Oliveira-Sales et al., 2009; Carillo et al., 2012). Ten minutes after the bicuculline injection (BIC condition), the activity of both nerves was recorded for 10 more minutes.

The acquired signals were analyzed offline using the appropriate software (Spike Histogram – PowerLab – AD Instruments, Australia). The integrated voltage responses of bursting rSNA and sSNA were expressed in arbitrary units (AUs) as the change (Δ) from the baseline (BAS) values obtained immediately before each test.

At the end of the experiments, the background noise of sympathetic nerve activity was determined by hexamethonium bromide administration (30 mg/kg, intravenously). At this dose, hexamethonium blocks the sympathetic vasomotor activity in rats, allowing for the subtraction of background noise and the quantification of only the sympathetic postganglionic activity (Touw et al., 1980).

The precision of the microinjection infusion was confirmed by the administration of Evans Blue (2% in 100 nl) into the area. After all proceedings, rats were euthanized with a lethal dose of urethane.

All experimental procedures were conducted in accordance with the Guide for the Care and Use of Laboratory Animals (8th edition, National Academies Press).

### 2.6. Signal pre-processing

All data sets were down-sampled to 1,000 Hz of sampling rate and were processed using an IIR notching comb filter to remove 60 Hz and harmonics. Additionally, a bandpass IIR filter was designed with an order of 12 and bandpass ripple 2 dB of 1 and 200 Hz.

### 2.7. Envelope signals

**FIGURE 1:**
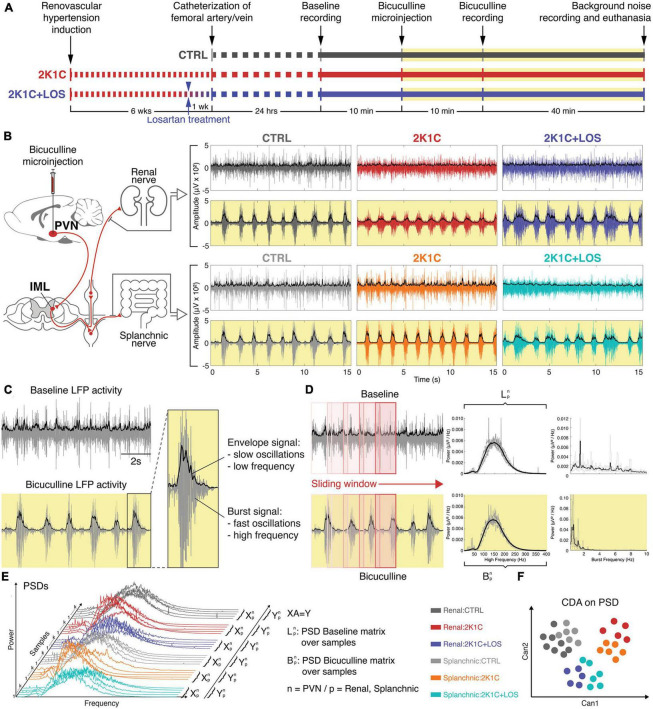
Methods. **(A)** Timeline of the hypertension induction, recordings and bicuculline injection. **(B)** Left: schematic of the projections from the paraventricular nucleus of the hypothalamus (PVN) to the intermediate lateral horn of the spinal cord (IML) and from the postganglionic neurons to the renal and splanchnic territories. Right: recordings of both renal and splanchnic sympathetic nerves activity from baseline (white background) and after bicuculline injection (yellow background). **(C)** Representative example of the envelope signal at slow oscillations and the filtered signal of burst activity at high oscillations. **(D)** Schematic of the sliding window analysis and the resulting spectrogram from baseline (white background) and after bicuculline injection (yellow background). **(E)** Schematic of the generation of the canonical discriminant analysis (CDAs) from the power spectral density (PSDs) distributions. **(F)** Illustrative example of a scatter plot generated from the CDA with two canonical variables. **(B,E,F)** Renal nerve: CTRL group, dark gray; 2K1C group, red; 2K1C + LOS group, dark blue. Splanchnic nerve: CTRL group, light gray; 2K1C group, orange; 2K1C + LOS group, turquoise.

All recordings were enveloped in order to highlight the slow frequencies related to bursts yielded by the injection of bicuculline (BIC) and the slow virtual frequencies of the BAS activities. The upper limit of the enveloped signals was calculated using the MATLAB^®^ function “envelope()” by implementing the root-mean-square (RMS) envelopes of all 15 recordings, using a sliding window of 50 samples. The analytic envelope signal associated with the input time series was calculated using the Hilbert transform (MATLAB Envelope, n.d.). Figure 1 shows how the sympathetic recordings were assessed.

### 2.8. Power spectrum density

The (local field potential) LFP recordings were analyzed according to their power spectrum in the frequency domain. The spectrograms were generated to evaluate the power of the frequencies over time, where the power spectrum density (PSD) per animal (*n* = 5 per group, with mean PSD estimated for each group), nerves (renal and splanchnic) and condition (BAS and BIC) were calculated by sliding a temporal window along each recording using MATLAB^®^ function “pwelch().” The time-window length was heuristically selected, considering 0.5% of the total signal, and 20% of overlap for the sliding steps, in order to maximize temporal and frequency resolutions.

### 2.9. Canonical discriminant analysis

The canonical discriminant analysis (CDA) was applied on the PSDs attempting to discriminate the shape of their power density distribution (Guang and Maclean, 2000; Reyes-Garcia et al., 2018; Pinheiro et al., 2020), using the MATLAB^®^ function “manova1(X, group),” where X is a matrix given by trials vs. power/frequency and group is a vector with labels associated with each one of the six conditions. CDA can reduce the space dimension of a set of variables by identifying orthogonal vectors (canonical variables) in the dependent space, which explains the greatest possible between-group variation (Biecek, 2015). The canonical variables CANs are linear combinations of the original variables that are chosen to maximize the separation between groups. CAN1 is the linear combination of the X columns that has the maximum separation among groups, with the most significant F statistic in a one-way analysis of variance. CAN2 has the maximum separation among clusters in the orthogonal space to CAN1 given the *F* statistic criteria and CAN3 has the maximum separation among clusters in the orthogonal space to CAN2 given the *F* statistic criteria.

All the PSDs calculated from each animal and nerve for both conditions (BAS and BIC) were grouped as shown in Figure 1E in order to evaluate if they had distinct and characteristic power shapes in specific frequency ranges. Here, we consider that each frequency range corresponds to the set of independent variables. In this way, if the shape of the power density distribution has enough information to discriminate each group described by the categorical dependent variable, the CDA will be able to identify and separate them (Figure 1F).

To highlight the similarities and differences between the clusters generated by the CDA, we also plotted the arrangement and relationship between the centroids of each cluster. This analysis allowed us to more accurately assess the effect of bicuculline and losartan on the different conditions and nerves.

### 2.10. Statistical analysis

Kolmogorov–Smirnov test was applied to verify the probability of samples coming from a normal distribution. Representations for statistical comparisons were made using mean/median ± CI for parametric and non-parametric data, respectively. To verify statistical differences associated with each nerve and condition, multivariate and bivariate tests were used, such as *n*-way ANOVA and *t*-test (or Kruskal–Wallis and Mann–Whitney test for non-parametric distributions).

Kruskal–Wallis test was performed to analyze the differences in power and synchronicity (through interval inter-bursts). For all analyses, a significance level α = 5% was used, and all signal processing and statistical analyses were performed using MATLAB^®^ predefined functions and homemade scripts (version 9.2.0 R2018a, Mathworks Inc., MA, USA).

## 3. Results

### 3.1. The systemic treatment with losartan did not revert the parameters altered by bicuculline injection into the PVN in the 2K1C animals

**FIGURE 2:**
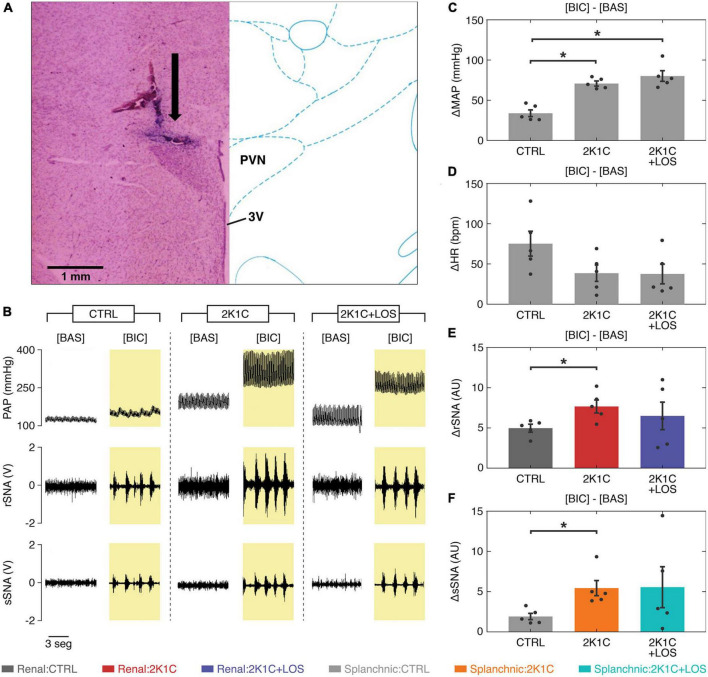
Systemic treatment with losartan did not revert the altered parameters in the 2K1C animals after bicuculline. **(A)** Representative histological image: arrow indicates the site of microinjection into the parvocellular region of the PVN; 3V, third ventricle. **(B)** Representative traces of pulsatile blood pressure (PAP), renal sympathetic nerve activity (rSNA), and splanchnic sympathetic nerve activity (sSNA) at baseline and after bicuculline injection. **(C)** The increase in the mean arterial pressure (MAP) levels after bicuculline injection from baseline is higher in both hypertensive groups (2K1C and 2K1C + LOS) as compared to the control group (CTRL) regardless of the losartan treatment. **(D)** The increase in heart rate was not statistically significant between groups. **(E,F)** The increase in both renal and splanchnic nerves activity (rSNA and sSNA, respectively) after bicuculline injection from baseline levels is significantly higher in the 2K1C group as compared to the CTRL group, but not to the 2K1C + LOS group. **(C–F)** Values plotted as mean ± SEM. *p < 0.05 (one-way ANOVA followed by Bonferroni’s *post-hoc* test). **(E,F)** Renal nerve: CTRL group, dark gray; 2K1C group, red; 2K1C + LOS group, dark blue. Splanchnic nerve: CTRL group, light gray; 2K1C group, orange; 2K1C + LOS group, turquoise. BIC, bicuculline condition; BAS, baseline condition.

The systemic administration of losartan (2K1C + LOS group) was not enough to significantly revert the increased response in the MAP (Figure 2C) and the decreased response in the HR (Figure 2D) between bicuculline and baseline conditions observed in the hypertensive animals (2K1C group). Likewise, it had no significant effect in reducing the over-activation of the renal (rSNA, Figure 2E) and splanchnic sympathetic nerves (sSNA, Figure 2F) after bicuculline injection into the PVN in the 2K1C group.

### 3.2. Bicuculline increases the burst envelope power at the 0–4 Hz band with no effect on the high frequency burst activity

**FIGURE 3:**
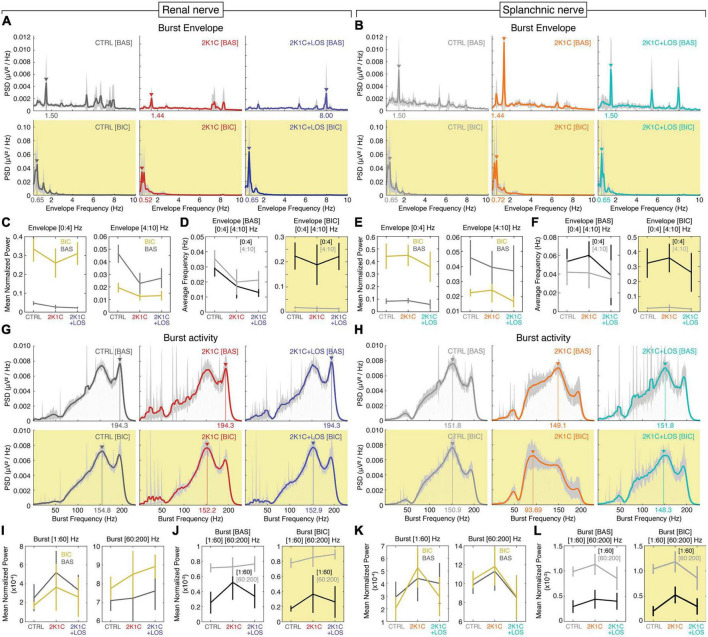
Bicuculline increases the burst envelope power at the 0–4 Hz band with no significant changes in the burst frequency power at the 60–200 Hz band. Mean PSD (power spectral density) spectrograms of the low frequency envelopes of the rSNA **(A)** and sSNA **(B)** and of the high frequency bursts of the rSNA **(G)** and sSNA **(H)** at baseline (top rows, white background) and after bicuculline injection (bottom rows, yellow background). Confidence intervals (CI) values in light gray. Mean power of the rSNA **(C)** and sSNA **(E)** at the 0–4 Hz (left) and 4–10 Hz (right) frequency bands and of the rSNA **(I)** and sSNA **(K)** at the 1–60 Hz (left) and 60–200 Hz (right) frequency bands at baseline (BAS) (dark gray line) and after bicuculline injection (yellow line). Comparison of the average frequency of the maximum power of the rSNA **(D)** and sSNA **(F)** between 0–4 Hz (dark gray line) and 4–10 Hz (light gray line) bands at baseline (left) and after bicuculline injection (right) and of the rSNA **(J)** and sSNA **(L)** between 1–60 Hz (dark gray line) and 60–200 Hz (light gray line) bands at baseline (left) and after bicuculline injection (right). **(A,B,G,H)** Renal nerve: CTRL group, dark gray; 2K1C group, red; 2K1C + LOS group, dark blue. Splanchnic nerve: CTRL group, light gray; 2K1C group, orange; 2K1C + LOS group, turquoise. **(A–L)** BAS, baseline condition; BIC, bicuculline condition.

At visual inspection, two main frequency bands stand out in the electrophysiological activity of the two nerves during baseline: 0–2 and 6–8 Hz (Figures 3A, B), with the 2K1C + LOS group exhibiting higher power peaks at 5 and 8 Hz than the other groups. After bicuculline administration, these peaks disappear and the power distribution shifts to two main components, one around 0.5 Hz and a weaker one around 1.5 Hz, which is in accordance with previous findings (Kenney et al., 2001) suggesting that the bicuculline injection in the PVN induces a highly synchronized pattern of low frequency activity in both nerves.

The bicuculline microinjection increased the bursting activity envelope power of the lower frequencies (0–4 Hz) in both nerves and all three groups, without significant differences among groups (Figures 3C, E, left panels). On the other hand, it had the opposite effect on the frequency band between 4 and 10 Hz, although not significant in the 2K1C group in both nerves and in the 2K1C + LOS group in the splanchnic nerve (Figures 3C, E, right panels).

There was no statistical difference between the 0–4 and the 4–10 Hz bands at baseline (Figures 3D, F, left panels). Bicuculline injection, however, promotes a clear separation between the two bands by increasing the 0–4 Hz power and decreasing the 4–10 Hz power (Figures 3D, F, right panels). This statistical effect corroborates the patterns of spectral distribution observed in Figures 3A, B.

With respect to the high frequency bursts, no major differences can be noted at visual inspection among the spectral patterns of the renal and splanchnic nerve activity before and after bicuculline injection. The power distribution is clearly concentrated at frequencies above 60 Hz in both nerves and all three groups, with peaks at around 150 and 190 Hz, which can be seen at a visual inspection of the PSDs (Figures 3G, H). The only exception is the splanchnic nerve activity in the 2K1C group after bicuculline injection that presents a shift in the power peak to around 90 Hz.

No significant differences were found in the mean power of renal or splanchnic nerve activity in any of the three groups between baseline and bicuculline in the high frequency bursting activity (Figures 3I, K). Comparisons between frequency ranges, however, confirmed that the 60–200 Hz power is higher than the 1–60 Hz in all groups, except for 2K1C in the renal nerve, without significant effect of the bicuculline injection in this pattern (Figures 3J, L).

### 3.3. Clustering analysis using canonical discriminant analysis revealed specific frequency patterns on each nerve for each of the three groups (CTRL, 2K1C, and 2K1C + LOS) and two conditions (BAS and BIC)

By means of the CDA we performed a joint multivariate analysis considering all the three groups (CTRL, 2K1C, and LOS), both nerves activities (renal and splanchnic) at baseline and after bicuculline administration at the high frequency range of the bursting activity as well as the low range of the envelope oscillations. The advantage of this analysis is that it provides a statistical global view highlighting the homogeneity among animals and heterogeneity among groups.

**FIGURE 4:**
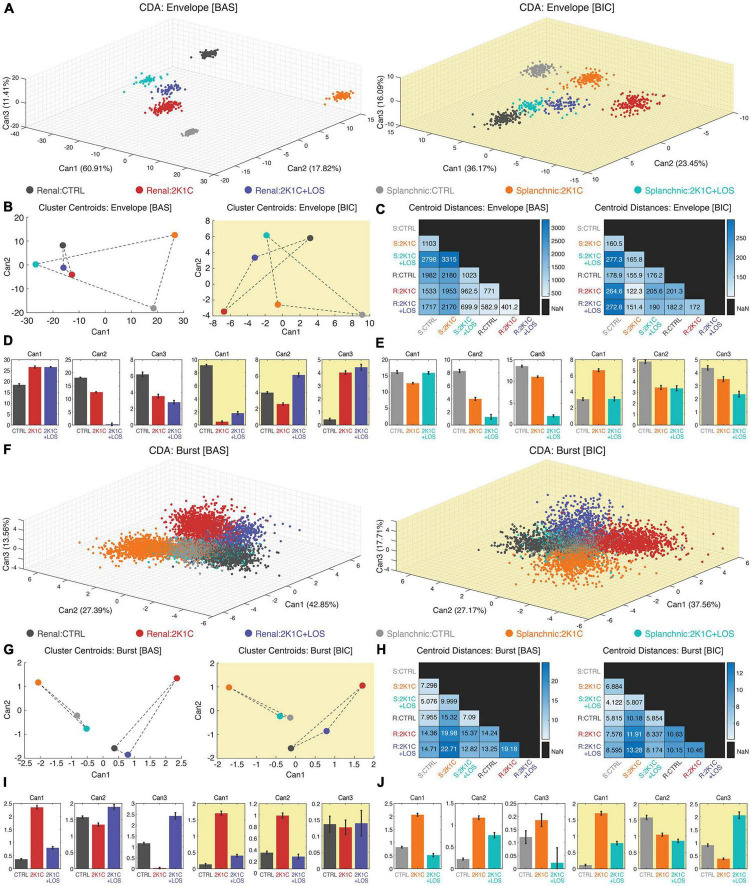
Canonical discriminant analysis (CDA) revealed specific frequency patterns for each nerve and each group in each condition, in both envelope and burst oscillations. Three-dimensional scatter plot of the three first canonical variables from the clustering analysis using CDA of both rSNA and sSNA envelope oscillations **(A)** and high frequency burst oscillations **(F)** at baseline (left, white background) and after bicuculline injection (right, yellow background). It is possible to notice the clusters separation at visual inspection. **(B,G)** Clusters’ centroid plotted using the first and second canonical variables (CAN1 and CAN2), that discriminated between each of the groups. **(C,H)** Matrix of the centroid distances for each pair of groups’ clusters from baseline (left) and after bicuculline injection (right). Confidence intervals of the first three canonical variables for the rSNA **(D,I)** and sSNA **(E,J)** at the baseline (left graphs, white background) and after bicuculline injection (right graphs, yellow background). **(D,E,I,J)** Values plotted as mean ± SEM. Confidence intervals of average: CI (m, 95%); one-way ANOVA followed by Bonferroni’s *post-hoc* test. **(A–J)** Renal nerve: CTRL group, dark gray; 2K1C group, red; 2K1C + LOS group, dark blue. Splanchnic nerve: CTRL group, light gray; 2K1C group, orange; 2K1C + LOS group, turquoise. **(A–C,F–H)** BAS, baseline condition; BIC, bicuculline condition. **(C,H)** R, renal; S, splanchnic.

The cluster analysis was carried out with the first three canonical variables from the CDA using the PSDs shapes as the main feature (Figure 4). The clusters are formed by the first three canonical variables values plotted against its respective axis in a three-dimensional space. Each cluster represents the spectral activity distribution across the frequency range of sympathetic vasomotor activity for a specific group. The spectral patterns of all five animals were included, meaning that the statistical effects take into account all three groups (CTRL, 2K1C, and 2K1C + LOS) and two nerves (renal and splanchnic) simultaneously for each frequency band (low frequency envelope oscillations, Figures 4A–E; high frequency bursts, Figures 4F–J).

Regarding the envelope oscillations analysis, all clusters are clearly separated in the three-dimensional space and statistically different at the sympathetic baseline activity (Figure 4A, left panel). This statistical trend can also be seen after the administration of bicuculline in the PVN (Figure 4A, right panel), although the clusters are spatially closer at visual inspection compared to the baseline condition, suggesting that the disinhibition of PVN tends to reduce the differences of sympathetic oscillatory patterns among groups and conditions.

This observation is supported by the analysis of clusters’ centroids distances. The centroid position is calculated by the average coordinates of all points that form a cluster. Since each cluster represents the spectral activity distribution for a specific group and sympathetic nerve, the distance between two centroids reflects the degree of similarity between their rhythmic patterns. In other words, changes in the neural activity pattern of a given group/nerve can be detected by analyzing the distances between clusters in the frequency space. After bicuculline injection in the PVN, the centroids’ relative distance values dropped down up to 20 times (Figure 4C). Interestingly, the envelope oscillating patterns of the renal nerve are more similar among the three conditions than the splanchnic nerve activity at the baseline condition (Figure 4B). It is noteworthy that the 2K1C + LOS group (blue dot) is almost equidistant from the CTRL group and the 2K1C group in both sympathetic nerves and conditions, except for the renal nerve at baseline, suggesting that it induces an oscillatory effect totally different from the other two groups. Additionally, Figure 4D (renal nerve) and Figure 4E (splanchnic nerve) show the confidence interval comparisons among the canonical variables averages of each nerve for the three groups before and after bicuculline.

The same set of analysis was carried out considering the whole frequency range to include the high frequency oscillations of the sympathetic bursting activity. The cluster analysis with the first three canonical variables from the CDA included all animals from each group and both sympathetic nerves. At visual inspection, it can be seen that at baseline condition the parameters are still well clustered (Figure 4F, left panel), although not as separated as for the envelope oscillations (Figure 4A, left panel). Bicuculline injection in the PVN also seems to have decreased the distance among clusters (Figure 4F, right panel), although apparently to a less extent than for the envelope oscillations (Figure 4A, right panel).

Analysis of the clusters’ centroid distances, however, revealed a linear separation among groups in both sympathetic nerves and both conditions (before and after bicuculline), which can be noticed by the absence of intersection between the triangles formed by the lines joining the groups of each nerve (Figure 4G). Furthermore, it is noteworthy that the centroids of CTRL and 2K1C + LOS groups are remarkably closer in the high frequency analysis than in the envelope oscillations in both conditions, which is true even if we consider both sympathetic nerves together (Figure 4G). In other words, the four independent sympathetic nerve recordings (Renal in CTRL, Renal in 2K1C + LOS, Splanchnic in CTRL and Splanchnic in 2K1C + LOS) share relatively similar oscillation patterns of activity that are, in turn, rather distinct from the hypertensive groups, which is also supported by the statistical effects among groups in the confidence interval comparisons (Figures 4I, J). Thus, the blockade of AT1 receptors by losartan seems to act differently over the high frequency bursts and the envelope oscillations, with a more prominent effect in recovering a normotensive pattern only in the high frequency bursts.

Additionally, the bicuculline injection into the PVN has a milder effect over the high frequency bursts in decreasing the distances among clusters’ when compared to the envelope oscillations (Figure 4H). Hence, although the bicuculline administration in the PVN seems to have a general effect of increased oscillatory synchronization, this effect is more prominent over the envelope oscillations. The PVN disinhibition triggered by bicuculline seems to entrain the structural properties of the circuits of each pathway with some kind of rhythmic marker, which could explain the rhythmic signature of each one.

## 4. Discussion

In the present study, we investigated the role of the PVN on the vasomotor sympathetic activity regulation by disturbing its inhibitory tonus with the injection of the GABAergic antagonist bicuculline, recording the renal and splanchnic nerves activity and comparing sympathetic vasomotor discharges pre and post microinjection of the agent. Subjects were control and Goldblatt hypertensive rats, with one group of the latter being systemically treated with losartan, an antagonist of AT1 receptors with known antihypertensive effects. To the best of our knowledge, this is the first categorical demonstration that spectral parameters of the sympathetic vasomotor activity carry specific information to different territories that can be distinguished in both healthy and pathological conditions.

Our findings support the notion that the mean global power is a rather limited parameter to distinguish among the sympathetic nerve activity of control, hypertensive and hypertensive treated animals. Mean global power was only effective to distinguish between activity above and below 60 Hz (Figures 3J, L, left panels) and between conditions (baseline and bicuculline) in the range below 4 Hz (Figures 3C, E, left panels). In other words, mean global power is unable to yield refined information that distinguishes between groups. These results also support the idea that frequency and amplitude of the sympathetic discharges are independently modulated by the PVN.

The postganglionic sympathetic nerve activity is organized in bursts of high frequency action potentials between 100 and 150 Hz (Figures 3G, H, upper panels) that repeat in a periodic manner generating a rhythmic pattern of oscillatory steady state activity (Barman, 2020). The periodic pattern itself generates a virtual envelope frequency between 7 and 10 Hz (Figures 3A, B, upper panels). We therefore adopted an analysis approach dividing the recorded signal in two encoding mechanisms: high frequency bursts and low frequency envelope oscillations (Figure 1C).

Using the shapes of the PSDs of the recorded signals (and therefore their spectral distribution) as the main feature for the CDA analysis, we found a highly clustered pattern of spectral organization among groups and sympathetic nerves for the envelope oscillations at baseline (Figure 4A, left panel), suggesting that the sympathetic vasomotor discharges have a highly specific spectral composition either under normal, hypertensive or treated conditions.

Since each cluster represents the distribution of spectral activity across different frequencies for a specific physiological condition and sympathetic vasomotor nerve, changes in the distances between these clusters indicate direct changes in their rhythmic patterns. In other words, alterations in the patterns of neural activity can be observed by analyzing the distances between clusters in the frequency space.

Bicuculline microinjection into the PVN dramatically decreased the distance among clusters’ centroids for the envelope oscillations, but not enough to bring them together (Figure 4C). Since the clusters represent the nerve activity’s spectral features, this result means that local GABAergic antagonism increased the similarity of the rhythmic patterns among groups and sympathetic nerves. It also changed the power distribution over the frequency range, flattening the activity above 2 Hz and concentrating the power spectrum in a typical oscillation pattern around 1.5 Hz (Figures 4A, B).

Although not as separated as in the envelope oscillations, the high frequency clusters also revealed distinct sympathetic patterns of spectral organization, especially above 60 Hz. The bicuculline microinjection into the PVN had a much softer effect in decreasing the clusters’ centroid distances than it had in the envelope oscillations.

Hence, these results suggest that the GABAergic neurons of the PVN play an important role in the temporal and spectral organization of the postganglionic sympathetic nerves bursting activity and in the differentiation of the spectral features coded in the reciprocal communication between them and the CNS. The absence of inhibitory modulation from the PVN has a milder effect over the higher frequencies, but tends to converge the slow envelope oscillations of all nerves and groups into more similar spectral patterns. The high frequency activity is therefore probably generated in cardiovascular nuclei other than the PVN. Nevertheless, they seem to be temporally organized by the slower envelope oscillations. However, once the patterns of sympathetic discharges are still highly distinct after bicuculline injection, it is highly unlikely that the PVN is the only structure generating the envelope frequency to distinct target territories. On the contrary, the spectral and temporal pattern of the slow envelope oscillations might be the result of the integrated activity of the PVN and other central nuclei through some cross-frequency mechanism. One of the main types of these mechanisms is the phase-amplitude coupling, in which the amplitude of a high frequency oscillation is coupled to the phase of a low frequency oscillation, a wave morphology that resembles the bursting pattern of the renal and splanchnic sympathetic activity. Phase-amplitude coupling has been extensively described in the CNS as the amplitude of a high frequency oscillation, such as a gamma (30–100 Hz), being coupled to the phase of a low frequency oscillation, such as delta (1–4 Hz) or theta (4–8 Hz) (Lisman and Jensen, 2013). Theta-Gamma and Delta-Gamma coupling have been functionally associated with the long-range communication between hippocampus and prefrontal cortex respectively in declarative memory tasks (Tort et al., 2009; Fell and Axmacher, 2011; Lisman and Jensen, 2013; Colgin, 2016) and working memory tasks (Lara and Wallis, 2015; Ga̧gol et al., 2018), suggesting that this mechanism is involved in the formation and retrieval of memory traces.

One possible explanation for this mechanism is that the slow oscillations provide traveling waves of excitability at whose peaks action potentials are more likely to happen. Therefore, by creating periodic windows of firing opportunity, slow oscillations would work as a probabilistic mechanism of temporal organization of fast spiking local circuits across long range networks (Fries, 2005; Buzsaki, 2006; Tal et al., 2020). Phase coherence between the activity of two given circuits and slow wave oscillations would be the key feature for integration. Theta oscillations underlie the integration of a network composed by the lateral amygdala, the infralimbic area of the medial prefrontal cortex and the CA1 area of the hippocampus during retrieval of conditioned fear in a contextual fear conditioning paradigm (Lesting et al., 2011). During extinction, coupling between LA and CA1 decreases, but this process can be delayed if both sites are electrically stimulated with synched in phase theta bursts, but not with a phase shift of 180° between sites (Lesting et al., 2011). This result shows how crucial the temporal organization is for the functional long-distance coupling of different areas during a specific task. We hypothesize that a similar mechanism underlies the functional integration of brainstem, basal forebrain and spinal cord nuclei – such as the PVN, the rostral ventrolateral medulla (RVLM) and the preganglionic motor neurons in the intermediate lateral horn (IML) of the spinal cord – with feedback information coming from sensors as baroreceptors. Parallel projections from the PVN’s parvocellular subdivision target both the RVLM and the IML (Badoer, 2001). The RVLM, in turn, projects directly to the IML, composing a network through which the PVN can modulate the preganglionic activity both directly and indirectly. The number of PVN neurons targeting the RVLM, however, is seven times higher than the number of those targeting the IML (Shafton et al., 1998). Besides, around 30% of the PVN neurons targeting the IML also send collateral projections to the RVLM (Badoer, 2001). Thus, despite its direct projections to the IML, the PVN seems to modulate the preganglionic neurons activity – and, by extension, sympathetic nerve activity – mostly indirectly, through the RVLM connection. We believe that the distinct patterns of activity described in this work may be the key mechanism by which the PVN organizes the precise temporal activation of viscerotopic subdivisions of the RVLM (McAllen et al., 1995) and IML and their output to specific target territories through the sympathetic nerves activity.

The blockade of the AT1 receptors via systemic treatment with losartan did not fully restore the baseline levels of autonomic symptoms and vasomotor activity in the Goldblatt hypertensive rats (Figures 2C–F). Notwithstanding, AT1 receptors somehow modulate the power spectral distribution in both nerves’ activity, once there were significant differences between the spectral parameters of the group 2K1C + LOS in relation to the others in almost all comparisons in the CDA analysis (Figure 4). The fact that systemic treatment with losartan did not restore the MAP levels increased by the bicuculline microinjection into the PVN in 2K1C rats suggests that the changes in the sympathetic vasomotor discharges triggered by the local neurons disinhibition are not dependent on blood pressure alterations.

The losartan treatment effect on the envelope oscillations, however, followed quite different patterns between renal and splanchnic sympathetic activity, especially for the envelope oscillations (Figures 4A–E). This result can be explained by the fact that the sympathetic vasomotor activity is topographically controlled through differential coding by the CNS, that is, the activity pattern to the kidneys can have distinct intrinsic parameters than the splanchnic territory and vice-versa. In addition, with the fact that frequency and amplitude of vasomotor activity can be also independently controlled by the brain, it is not surprising that the vasomotor responses induced by the losartan treatment were different for the renal and splanchnic territories mainly with regard to the envelope oscillations, but not much to the high frequency activity (McAllen and Malpas, 1997). Nevertheless, it should be noted that the losartan treatment in the present study took place for only 1 week and it would be important to investigate whether the mechanisms generating the sympathetic vasomotor activity would be modified by a longer treatment.

The effective impact of the PVN disinhibition in the peripheral organs was not addressed in this study and remains to be investigated. Notwithstanding, it is interesting to point out that, even in the face of a significant decrease in the frequency range (and thus the number of bursts per second), the pattern of adrenergic discharges generated by the PVN disinhibition was still able to raise the blood pressure in all groups. As mentioned before, the CNS can control the amplitude and frequency of ongoing sympathetic activity independently (McAllen and Malpas, 1997). However, the negative correlation between frequency and amplitude with impact on the physiological parameters is not necessarily to be considered dichotomic, as it can be interpreted as a power shift to a lower frequency range.

## 5. Conclusion

In summary, our results strongly suggest that frequency-encoded patterns are part of the mechanisms underlying sympathetic vasomotor discharges in both normal and hypertensive conditions and that these patterns are modulated by the inhibitory tonus of the PVN. If this stands true also for the other nuclei composing the complex network that controls sympathetic motor activity remains to be investigated. Nevertheless, the finding that the spectral features of sympathetic nerve activity carry relevant information that can distinguish between normotensive and hypertensive individuals is of major relevance for future basic and clinical studies regarding hypertension treatment approaches. Additionally, although it was not possible to dissociate the intrinsic effects of the virtual envelope oscillations over the high frequency bursts, the isolated analysis of the envelope oscillations suggested a possible amplitude-frequency coupling effect. To better understand its dynamics and role in the encoding and transmission of information in each nerve and condition, further analyses are still needed. Power alterations in the sympathetic nerve activity triggered by changes in the PVN GABAergic tonus are long known. The finding that it is also involved in the frequency patterns organization suggests that PVN GABAergic neurons might play a much more complex role in the temporal organization control of sympathetic discharges. The GABAergic system might as well become a relevant target for pharmacological approaches to hypertension treatment.

The results improve our understanding of the intricate mechanisms involved in the sympathetic vasomotor overactivation in conditions such as arterial hypertension and renal failure. In addition, we hope that the signal analysis approach used in this study might contribute to the progress of the field with a new set of powerful tools and techniques.

## Data availability statement

The data that support the findings of this study are not openly available due to reasons of sensitivity and are available from the corresponding author upon reasonable request.

## Ethics statement

The animal studies were approved by the Ethics in Research Committee of the Escola Paulista de Medicina – Universidade Federal de São Paulo (protocol no. 8724270715/15). The studies were conducted in accordance with the local legislation and institutional requirements.

## Author contributions

RC, MM, CS, and JF: conceptualization and writing. RC: project coordination and funding. JF and RC: project supervision. MM: data acquisition. CS: figure preparation. JF: data analysis. All authors read and reviewed the manuscript.
